# Complete Genome Sequence of the Clinical Strain *Acinetobacter baumannii* R2090 Carrying the Chromosomally Encoded Metallo-β-Lactamase Gene *bla*_NDM-1_

**DOI:** 10.1128/genomeA.01008-15

**Published:** 2015-09-10

**Authors:** Thomas Krahn, Daniel Wibberg, Irena Maus, Anika Winkler, Patrice Nordmann, Alfred Pühler, Laurent Poirel, Andreas Schlüter

**Affiliations:** a Center for Biotechnology, Bielefeld University, Bielefeld, Germany; b Medical and Molecular Microbiology Unit, Department of Medicine, Faculty of Science, University of Fribourg, Fribourg, Switzerland

## Abstract

*Acinetobacter baumannii* is an emerging human pathogen causing nosocomial and community-acquired infections. Here, we present the complete genome sequence of the clinical *A. baumannii* strain R2090 carrying the metallo-β-lactamase gene *bla*_NDM-1_ in its chromosome within the transposon Tn*125*.

## GENOME ANNOUNCEMENT

*Acinetobacter baumannii* is a widespread serious pathogen (1) causing nosocomial infections (2). Also, community-acquired infections initiated by this pathogen feature high mortality rates (3). In this study, we present the complete genome sequence of the strain R2090, representing a community-acquired *A. baumannii* sequence type.

The strain R2090 was recovered from a rectal swab from a patient hospitalized in an Egyptian hospital. It was classified as belonging to the species *A. baumannii*, since its 16S rRNA gene sequence is identical to the corresponding sequence of the *A. baumannii* type strain ATCC 19606 (4).

For the establishment of the *A. baumannii* R2090 genome sequence, purified chromosomal DNA was used to construct an 8-kb mate pair sequencing library (Nextera mate pair sample preparation kit, Illumina, Inc.). The sequencing approach on an Illumina MiSeq system yielded 3,108,361 sequence reads accounting for 699,271,346 bases sequence information. Thus, a 183-fold coverage was achieved for the 3.8-Mb *A. baumannii* genome. The obtained sequence reads were assembled using the GS De Novo Assembler software (version 2.8, Roche) (5, 6), which resulted in one scaffold composed of 57 contigs. Subsequently, an *in silico* gap closure approach followed by a PCR-based finishing strategy (7–9) was applied to complete the circular chromosome. Annotation of the 3,819,158-bp genome featuring a G+C content of 39.04% was performed within the GenDB 2.0 system (10) and resulted in the prediction of 3,601 coding sequences, 73 tRNA genes, and 6 *rrn* operons.

The genome of *A. baumannii* R2090 is highly related to the community-acquired *A. baumannii* strain D1279779 that was isolated from an indigenous Australian patient who suffered from a bacteremic infection (11). Both strains belong to sequence type 267. Calculation of the average nucleotide identity (ANI calculator [http://enve-omics.ce.gatech.edu/ani/]) confirmed close relatedness of both strains with an ANI value of 99.92%.

Comparative analyses of strains R2090 and D1279779 revealed differences in putative virulence determinants. In strain D1279779, genes encoding a type VI secretion system are missing (11). In contrast, strain R2090 carries at least 13 of 17 genes of the type VI secretion system cluster. Moreover, strain D1279779 harbors three type I pili systems putatively involved in adherence and biofilm formation (12), whereas two of these clusters featured insertions of transposable elements in strain R2090.

An ARG-ANNOT analysis (13) indicated presence of the metallo-β-lactamase (MBL) gene *bla*_NDM-1_ exclusively in the R2090 chromosome in addition to other putative resistance determinants present in both genomes. The gene *bla*_NDM-1_ encodes a broad-spectrum carbapenemase with the capability to inactivate all β-lactam antibiotics except aztreonam (14). As previously described, this gene is located on the transposon Tn*125* (15, 16). Occurrence of *bla*_NDM-1_ in strain R2090 is important with regards to the dissemination of this serious antibiotic resistance determinant among *A. baumannii* strains (11, 17).

Comparison of the R2090 genome to the genomes of other clinical *A. baumannii* isolates is expected to provide deeper insights into the development of multiresistant derivatives of this emerging human pathogen.

### Nucleotide sequence accession number.

The *Acinetobacter baumannii* R2090 genome sequence has been deposited in the EMBL/GenBank database (EBI, NCBI) under the accession number LN868200.
